# Sectional Anatomy Quiz - VII

**DOI:** 10.22038/AOJNMB.2021.55113.1381

**Published:** 2022

**Authors:** Nina Li, Rashid Hashmi

**Affiliations:** Rural Medical School, University of New South Wales (UNSW), Wagga Wagga, NSW, Australia

**Keywords:** Computed tomography, Sectional anatomy, Abdomen, Coeliac axis, Coeliac artery

## Abstract

This series involves a quiz pertaining to the identification of key anatomical landmarks and normal structures present at a given level on the computed tomography (CT) image. The current quiz demonstrates examples of normal and abnormal axial CT images at the level of origin of the coeliac artery. The representative image is subsequently followed by further images demonstrating various commonly encountered pathologies found at this level in clinical practice. In each image, readers are expected to identify highlighted anatomical structures and appreciate how given pathologies can alter the appearance of normal structures. This series aims to advance understanding of sectional anatomy and aid nuclear physicians in the interpretation of the CT component of single photon emission computed tomography (SPECT) and positron emission tomography (PET) studies.

## Introduction

**Figure 1:**
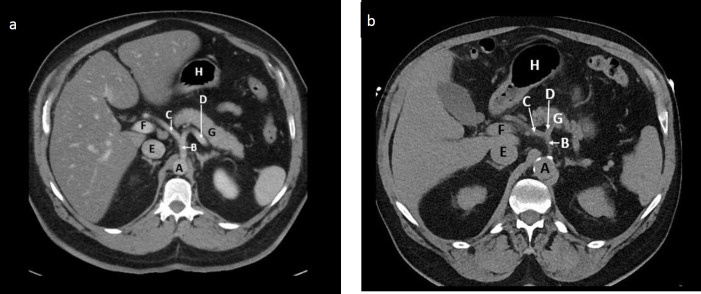
Contrast enhanced (**a**) and non-contrast (**b**) axial CT abdomen of two different patients at the level of the coeliac artery origin is shown. Identify the normal anatomical structures labelled A to H

**Answer**

 These images depict the upper abdomen at the level of the coeliac artery origin which is approximately at the level of the 12th thoracic vertebra (T12), inferior to the diaphragm.

A: Abdominal aorta with wall calcifications in (b)

B: Coeliac trunk

C: Common hepatic artery

D: Splenic artery 

E: Inferior vena cava (IVC)

F: Portal vein

G: Body of pancreas 

H: Stomach

**Figure 2:**
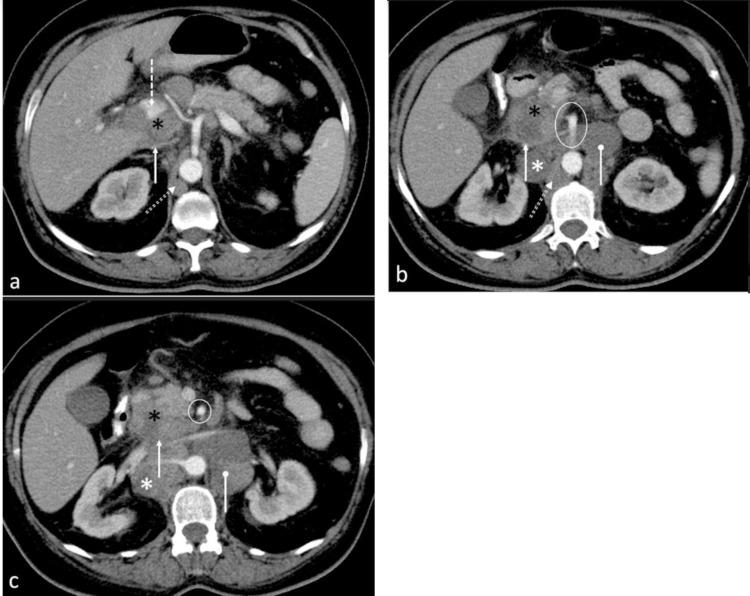
Contrast enhanced axial CT abdomen of a 43-year-old male at the level of the celiac artery origin (**a**) demonstrates an enlarged portacaval lymph node (black asterisk) which appears as a confluent soft tissue density mass between the portal vein (dotted white arrow) and IVC (solid white arrow). Images (**b **and **c**) acquired more caudally at and below the level of origin of the superior mesentery artery (circled) respectively, confirm compression of the IVC by lymph nodes. Note the enlarged retrocaval (white asterisk), para-aortic (oval arrow) and retrocrural (double dashed arrow) lymph nodes

**Figure 3:**
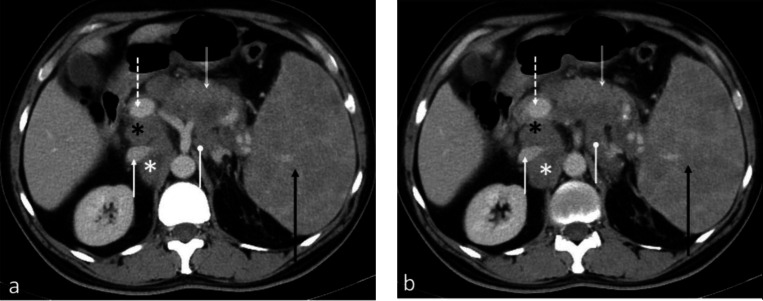
Contrast enhanced axial CT abdomen of a 46-year-old female with lymphoma shows enlarged retrocaval (white asterisk) and portacaval (black asterisk) lymph nodes. Portal vein and IVC are indicated by dotted and solid white arrows respectively. Also noted are an enlarged celiac lymph node (oval arrow) and spleen (black arrow). Heterogenous enhancement of the pancreas (double arrow) is suspicious of lymphomatous involvement. Image b is caudal to the image a. There is little consensus in literature on the best single measurement for assessing size of the spleen as its shape and configuration makes linear measurement difficult. The upper limit of normal adult splenic length is traditionally considered to be 12 cm

**Figure 4:**
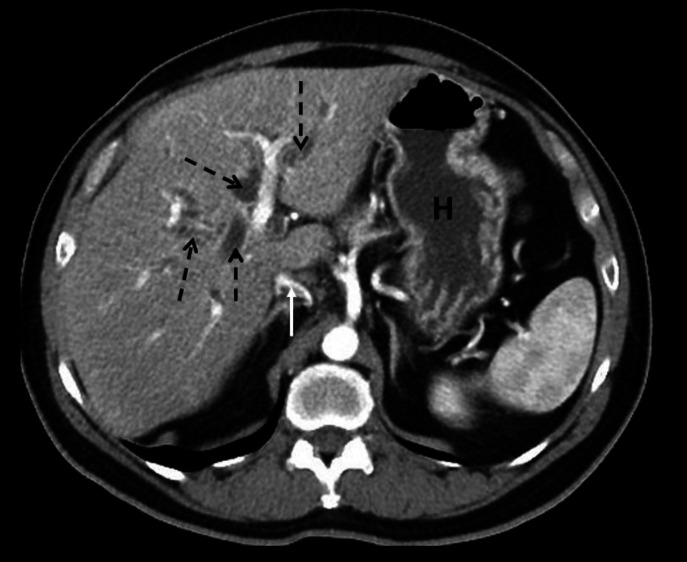
Contrast enhanced axial CT abdomen of a 46-year-old female illustrates intrahepatic biliary dilatation (dashed black arrows) appearing as linear or tubular and branching low-density areas in the liver. Biliary dilatation in this patient was due to an ampullary tumour which is not seen on the image. Note that normal intrahepatic bile ducts are small in calibre and only faintly seen on CT. They are considered to be dilated when they measure >3mm in diameter. It is important to appreciate normal variation in the calibre of the IVC (white arrow) which can be influenced by inspiratory effort and hydration status. It is also important to appreciate variation in the size and appearance of the stomach (**H**)

**Figure 5:**
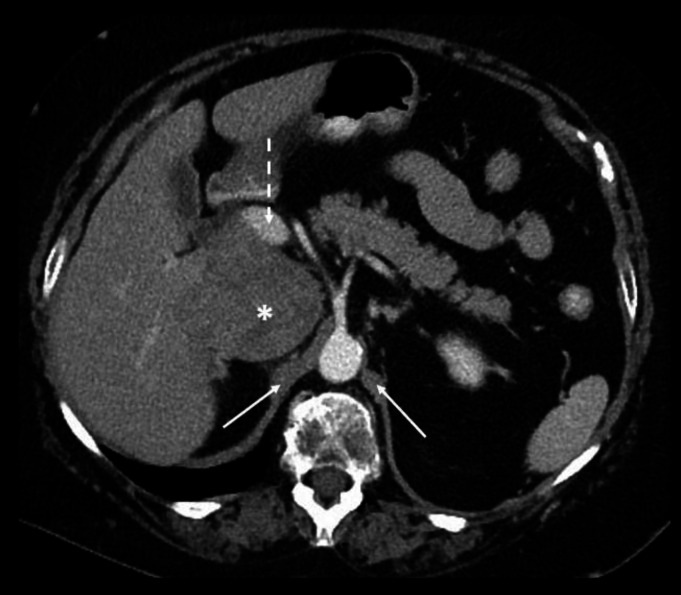
Contrast enhanced axial CT abdomen of a 80-year-old female demonstrates a large, expansile, soft-tissue density mass (asterisk) posterior to the portal vein (dashed white arrows). Review of other images show the mass to be separate from the liver but distinction between this mass and the IVC was not possible. The mass was diagnosed to be leiomyosarcoma arising from the IVC following surgery. It shall be remembered that the origin or epicentre of a mass may not be confidently determined on a single image and necessitates review of series of images in different orthogonal planes. Right and left crus of diaphragm are indicated by solid white arrows

**Figure 6:**
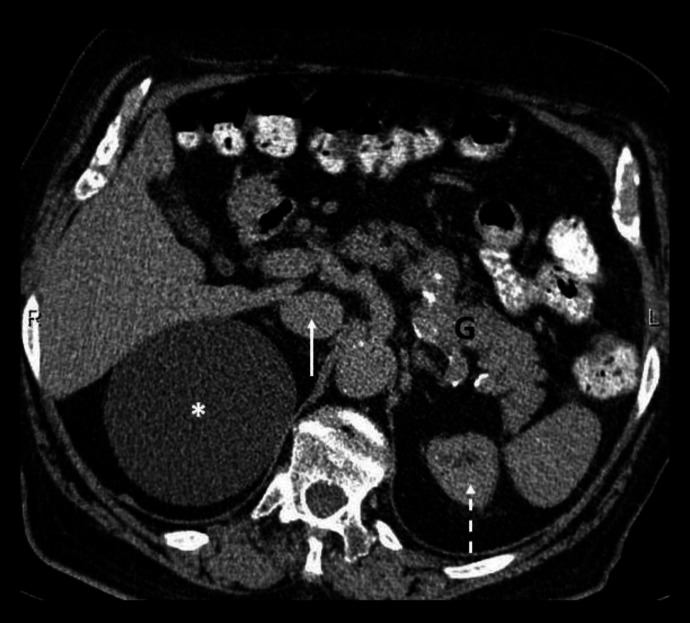
Contrast enhanced axial CT abdomen of a 56- year-old male shows a well-defined, homogenous, non-enhancing, low density lesion (asterisk) that on inferior images was found to be arising from the upper pole of the right kidney. The findings are suggestive of an exophytic simple renal cyst. A simple cyst, irrespective of its origin, is characterized by absence of calcification, septation, heterogeneity, nodularity, wall thickening or enhancement on post contrast images. Solid and dashed white arrows point to IVC and upper pole of the left kidney respectively. Specks of calcification seen close to pancreas (**G**) involve the tortuous splenic artery

**Figure 7:**
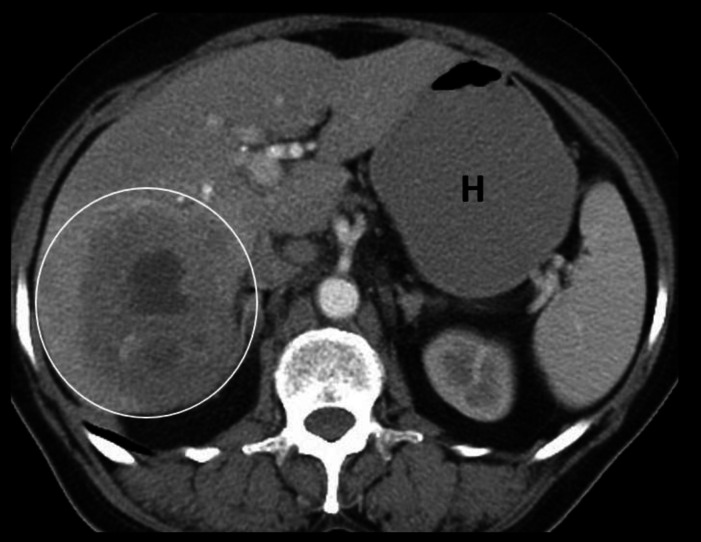
Contrast enhanced axial CT abdomen of a 64-year-old female with hepatic abscess (circled) shows an ill-defined, mix density lesion with heterogenous enhancement in the right lobe of the liver. It is to be noted that distinction between an abscess and a necrotic tumor solely on imaging features can be difficult and correlation with clinical features and other laboratory parameters is important. Note the Fluid filled distended stomach (**H**)

**Figure 8:**
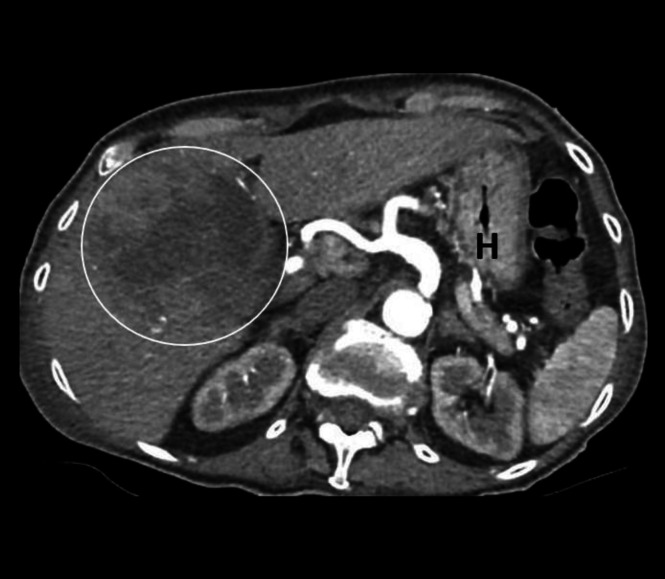
Contrast enhanced axial CT abdomen of a 90-year-old female with a history of hepatitis C infection, abdominal pain, abnormal liver function tests and elevated Alpha fetoprotein (AFP) shows a large, poorly defined lesion with areas of low attenuation (circled) in the right lobe of the liver. The lesion was subsequently diagnosed to be a necrotic hepatocellular carcinoma. Stomach (**H**) is collapsed and contains small amount of air. When collapsed, the wall of stomach appears thickened and should not be misinterpreted as pathological

**Figure 9:**
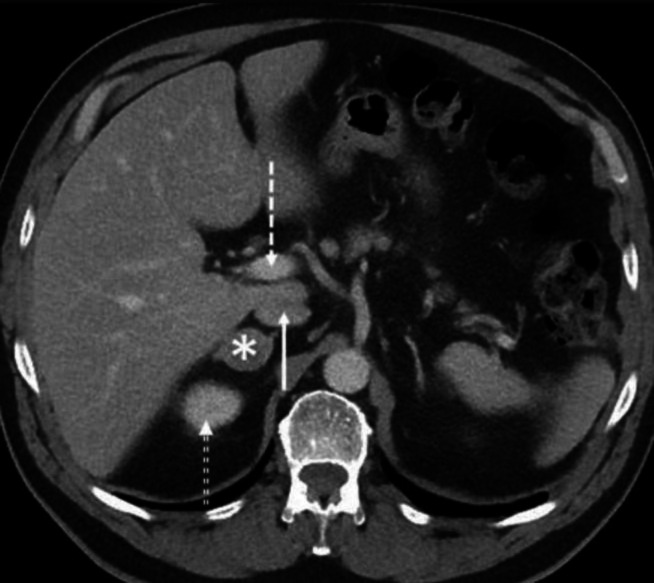
Contrast enhanced axial CT abdomen of a 70-year-old male who presented with clinical features suggestive of acute diverticulitis shows a well-defined, homogenous, low density lesion (asterisk) with mild enhancement in the right upper abdomen. The lesion is located posterior to the IVC (solid white arrow) and superior to the right kidney (double white arrow) suggesting it is originating from the right adrenal gland. As the density of lesion was measured to be 6HU, it most likely represents an adrenal adenoma. Adrenal adenoma is often found incidentally during abdominal imaging and has low density due to presence of intracytoplasmic lipid. On a non-contrast CT an adrenal lesion with attenuation of <10HU is considered highly specific for an adrenal adenoma. Note that CT attenuation of a lipid poor adenoma can be >10HU necessitating further workup (e.g., contrast enhanced CT or magnetic resonance imaging) for catherization of the lesion

**Figure 10:**
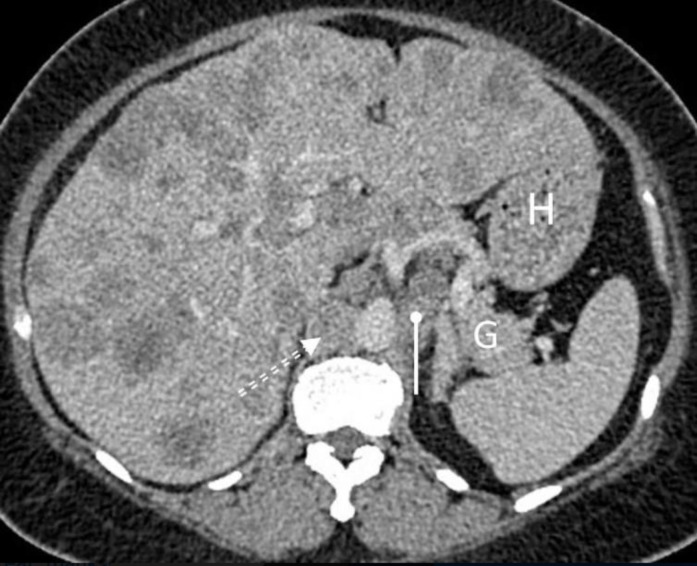
Contrast enhanced axial CT abdomen of a 56-year-old female with a history of carcinoma of the breast shows multiple, irregular low-density areas with heterogenous enhancement in the liver, suggesting metastasis. Note the enlarged retrocrural (double dashed arrow) and paraaortic (oval arrow) lymph nodes. Distal portion of body and tail of the pancreas (**G**) is seen adjacent to the splenic hilum. Stomach (**H**) is filled with food particles

**Points to remember**

- The coeliac trunk is a short vessel that splits into 3 branches: the common hepatic artery, splenic artery and left gastric artery. A bifurcation into the hepatosplenic trunk and left gastric artery is another anatomical variant, in addition to other branching patterns that may be present.
- The portal vein is formed by the union of the superior mesenteric vein and splenic vein at approximately the level of the second lumbar vertebra, posterior to the neck of the pancreas.
- The splenic artery can be identified by its origin from the coeliac trunk and tortuous pathway, initially running superior to the pancreas.
- Left lobe of the liver is separated by the falciform ligament and ligamentum venosum.
- Hepatocellular carcinoma (HCC) has variable

imaging appearance due to its various subtypes. 

 Acquisition of multi-phase contrast enhanced CT comprising of arterial phase (20-30 seconds after the injection of the contrast media), portal venous phase (50-60 seconds after the injection), and delayed phase (>120 seconds after the injection) is necessary for the correct characterization. A focal HCC characteristically shows early enhancement on the arterial phase followed by rapid washout on the portal venous phase becoming iso-dense or hypodense to the rest of the liver. On delayed phase, the HCC appears hypodense to rest of the liver.

- Degree of distension of the stomach influences thickness of its wall on CT. When fully or well distended, the wall of a normal stomach is about 2-3 mm in thickness in the body and 5-7 mm in the antrum. Wall thickness of 1 cm or greater of a distended stomach is abnormal.

